# Clonal architecture evolution in Myeloproliferative Neoplasms: from a driver mutation to a complex heterogeneous mutational and phenotypic landscape

**DOI:** 10.1038/s41375-023-01886-0

**Published:** 2023-03-31

**Authors:** Nabih Maslah, Lina Benajiba, Stephane Giraudier, Jean-Jacques Kiladjian, Bruno Cassinat

**Affiliations:** 1 Université Paris Cité, APHP, Hôpital Saint-Louis, Laboratoire de Biologie Cellulaire, Paris, France; 2 INSERM UMR 1131, Institut de Recherche Saint-Louis, Paris, France; 3 INSERM UMR 944, Institut de Recherche Saint-Louis, Paris, France; 4 Université Paris Cité, APHP, Hôpital Saint-Louis, Centre d’Investigations Cliniques, INSERM CIC 1427, Paris, France

**Keywords:** Genetics research, Cancer genetics

## Abstract

Myeloproliferative neoplasms are characterized by the acquisition at the hematopoietic stem cell level of driver mutations targeting the JAK/STAT pathway. In addition, they also often exhibit additional mutations targeting various pathways such as intracellular signalling, epigenetics, mRNA splicing or transcription. The natural history of myeloproliferative neoplasms is usually marked by a chronic phase of variable duration depending on the disease subtype, which can be followed by an accelerated phase or transformation towards more aggressive diseases such as myelofibrosis or acute leukemia. Besides, recent studies revealed important new information about the rates and mechanisms of sequential acquisition and selection of mutations in hematopoietic cells of myeloproliferative neoplasms. Better understanding of these events has been made possible in large part with the help of novel techniques that are now available to precisely decipher at the single cell level both the clonal architecture and the mutation-induced cell modifications. In this review, we will summarize the most recent knowledge about the mechanisms leading to clonal selection, how clonal architecture complexity can explain disease heterogeneity, and the impact of clonal evolution on clinical evolution.

## Introduction

BCR::ABL1-negative myeloproliferative neoplasms (MPNs) include essential thrombocythemia (ET), polycythemia vera (PV) and primary myelofibrosis (PMF). In these chronic hematological malignancies, the main short-term risk is the occurrence of thrombosis but a subset of patients may also evolve into secondary myelofibrosis, myelodysplastic syndrome or acute myeloid leukemia in the long run. However, the risk of long term evolution is heterogeneous between MPN subtypes: recent retrospective studies suggest that a high proportion of PV patients (up to 75% in 13 years) may experience progression to secondary myelofibrosis or AML [1], while in ET only a minority of patients experience clonal evolution and deterioration of MPN. The clinical course of MPNs is therefore characterized by a hitherto not fully understood nor accurately predicted inter-patient heterogeneity.

In the recent years, disease heterogeneity has been mainly linked to the diversity of genetic lesions found in patients’ hematopoietic stem cells (HSC). Indeed, MPNs represent a model of sequential acquisition of genetic abnormalities over time, allowing the study of the influence of environmental and intrinsic factors on tumor shape. Numerous studies have shown that precise genetic characterization of the disease can help to evaluate its prognosis [2] as the number and type of mutations are the main criteria considered to predict the outcome of patients. Indeed, recent prognostic scoring systems include the mutational pattern [3–5]. Dissecting the prognostic impact of diverse molecular markers allows a better understanding of the heterogeneity of tumor cells and demonstrates its predominant role in MPN evolution. Furthermore, implementation of new sequencing techniques at the single-cell level allows more precise characterization of complex molecular patterns associated with disease heterogeneity. Despite an improved understanding of the clonal architecture of MPNs over the past years, the mechanisms leading to clonal selection once the mutations are acquired remain poorly understood. In several types of cancers, a clear role of the microenvironment has been demonstrated in the selection of mutations. Specific clones harboring particular mutations may be selected due to inter-clone competition for nutrients or to the presence of an inflammatory environment. The drugs received during the chronic phase of the disease can also participate in clonal selection, which may be of particular importance in MPN patients who often require lifelong treatments. The aims of this review are to recapitulate the current knowledge of the different molecular lesions acquired in MPNs, highlight their impact on disease evolution and discuss the processes influencing their selection and expansion over time.

## Initiating mutations

**Fig. 1:**
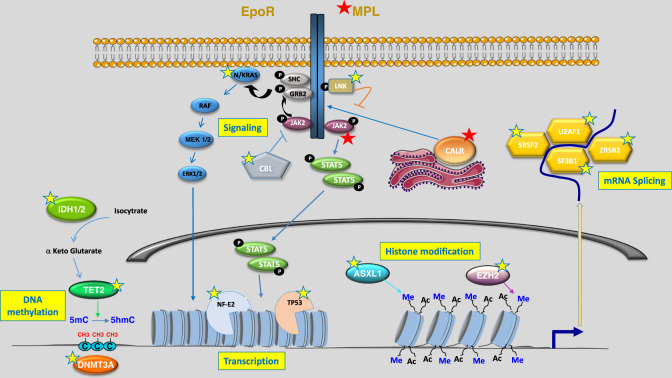
Main mutations identified in Myeloproliferative Neoplasms. MPNs are characterized by acquired mutations in genes controlling various cellular processes (yellow boxes), all involved in different steps of the gene expression regulation. The receptors of erythropoïetin (EPO-R) or of thrombopoïetin (MPL) are constitutively associated to the JAK2 kinase. The driver mutations initiating the disease (marked by red stars) target either *JAK2*, *CALR* or *MPL* genes, all three resulting in an activation of the JAK/STAT and RAS pathways. Additional mutations (marked by yellow stars) may be acquired before or after the driver mutation and can accumulate during disease evolution. Such mutations have been identified in genes involved in epigenetics (*TET2*, *ASXL1*, *DNMT3A*, *EZH2* or *IDH1/2*), mRNA splicing (*SRSF2*, *SF3B1*, *U2AF1, ZRSR2*), cell signalling (*NRAS*, *KRAS*, *CBL*, *SH2B3* coding for LNK protein) or factors regulating the transcription (*NFE2*, *TP53*). Of note, mutations targeting *PTPN11*, *NGAS*, *SETBP1*, *NF1* (signalling) or *RUNX1*, *ETV6*, *CUX1*, *STAG2*, *PHF6*, *BCOR*, *BCORL1* (transcription regulation) have also been reported (but less frequently) in MPN patients [2–4].

MPNs are characterized by the acquisition in the HSCs of mutations that activate not only the JAK2/STAT5 pathway but also STAT3 and, either in parallel or consecutively, PI3K-AKT and MEK-ERK pathways. These mutations, considered as initiators of the phenotype and called “drivers”, affect the *JAK2*, *MPL* or *CALR* genes (Fig. 1). An important aim has long been to determine the date of the acquisition of the mutations that are at the origin of MPNs development. Indeed, several studies have shown the possibility to detect mutations associated with myeloid malignancies in the blood of apparently healthy subjects, defining the notion of clonal hematopoiesis of indeterminate potential (CHIP), among which the JAK2^V617F^ mutation is quite frequently found [6, 7]. Furthermore, several studies reported the detection of JAK2^V617F^ mutations many years before the development of MPNs [8, 9] suggesting that this mutation might not immediately confer a proliferative advantage as intense as previously thought, and that it may take several years for the disease to develop. While one study found the JAK2^V617F^ mutation in cord blood [10], the acquisition of this mutation during childhood or the prenatal period has recently been suggested in several MPN patients [11, 12]. Similar findings have been reported for *CALR* mutations, although mathematical modelling also suggested that *CALR* mutations tend to be acquired later in life in comparison with *JAK2* mutations, potentially due to an increased proliferative advantage [13, 14]. In these studies, the latency between the acquisition of a driver mutation and the diagnosis of MPNs was several decades, suggesting that mutated clones may persist for a very long time before becoming overtly pathogenic. Regarding the initiating mutations, it is important to emphasize that the type of mutated gene influences the affected cell types. For instance, mutations in the *MPL* gene that encodes the thrombopoietin receptor will specifically affect cell types expressing this receptor during hematopoiesis (i.e., mostly megakaryocytic lineage), while mutations in *JAK2* will broadly alter the signaling of a variety of receptors expressed in almost all hematopoietic cell types. Therefore the consequences of each type of initiating mutation will differ in terms of deregulation of hematopoietic lineages and clonal and clinical evolution during the course of the disease [15].

## Additional mutations

On top of the driver mutations, MPN patients may also acquire mutations considered as “additional”, targeting genes involved in various cellular processes. A non-exhaustive list of most frequently affected genes is given in Fig. 1. The processes impacted by these mutations are all involved in the regulation of gene expression: intracellular signaling pathways, epigenetics (DNA methylation, post-translational modifications of histones), transcription factors, RNA splicing. Thus, since the beginning of the 2010s it appeared that MPNs were possibly oligoclonal diseases (with the coexistence of several molecularly distinct clones) rather than monoclonal pathologies with accumulation of mutations in a single founder clone [16, 17]. However, it was rapidly shown that the clonal origin of the mutations is complex as two types of patterns can be distinguished: on the one hand, patients who first acquire a mutation in a driver gene and then additional mutations, and on the other hand, patients who acquire driver mutations within cells that have already acquired a mutation in non-driver genes, most frequently in genes involved in CHIP such as *TET2* or *DNMT3A* [12, 18, 19]. Although the type of MPN and driver mutation have been correlated to different clinical outcomes, it appeared that additional mutations have significant impact on the prognosis of patients according to the disease type. For example, mutations affecting *ASXL1*, *EZH2*, *SRSF2* and *IDH1/2* genes are associated with a poor prognosis in PMF patients, defining a group of so-called high molecular risk (HMR) mutations [20], but mutations in the *TP53* [18, 21, 22], *NRAS*/*KRAS* [23, 24] and *NFE2* [25] genes have also been associated with a poorer outcome. In PV and ET spliceosome mutations have been shown to adversely affect overall survival (*SF3B1*, *SRSF2* in ET, and *SRSF2* in PV) and myelofibrosis-free survival (*U2AF1*, *SF3B1* in ET), while *TP53* mutations predicted the risk of leukemic transformation in ET [26]. Furthermore, it has been shown that the accumulation of mutations is in itself an adverse prognostic factor in MPNs since the number of HMR mutations at diagnosis is correlated with the risk of transformation whatever the MPN subtype [27, 28]. Based on these findings, several prognostic scores including molecular data have recently been proposed, in particular to predict the outcome of MF patients such as the MIPSS70, MIPSS70 +  [3, 4] or the MTSS for patients who undergo stem cell transplantation [5]. Confirmation of the deleterious role of co-occurrence of JAK2^V617F^ mutations with some of these mutations has been provided by animal models. For example, the transduction of JAK2^V617F^ in the bone marrow cells of *TP53* knockout mice induced the occurrence of leukemia in the recipient mice [21]. Similarly, while deletion of the *EZH2* gene on its own doesn’t induce a marked phenotype in mice, crossing *EZH2* knockout mice with JAK2^V617F^ transgenic mice results in accelerated onset of myelofibrosis and marked shortening of the lifespan compared to JAK2^V617F^ mice [29]. These two observations demonstrate that additional mutations expressed in the same cells along with JAK2^V617F^ alter cell fate and can accelerate the evolution of MPNs, confirming that clonal developments can be important milestones driving clinical progression. Importantly, the presence of additional mutations not only influences the clinical course of the disease but may also modify response to treatment. Indeed, the risk of developing resistance to hydroxyurea (HU) appears to be higher in patients with mutations in *TP53* or genes regulating RNA splicing [30] while mutations in the *RAS* pathway are more frequently associated with resistance to the JAK inhibitor ruxolitinib [23, 24]. Of note, besides being mutated, splicing factors may also exert altered function by undergoing posttranslational modifications. For example, it has been shown that modulation of these post-translationally modified splicing factors downstream of mutated JAK2 kinase had an impact on clonal persistence and progression [31]. Similar mechanisms have been explored for epigenetic modifiers such as KDM4C or JMJD2C in JAK2-mutated cells [32, 33].

## Clonality features of MPNs

**Fig. 2:**
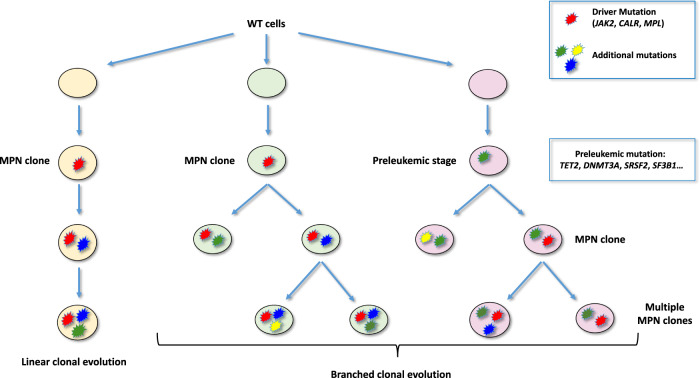
Different models of clonal evolution in Myeloproliferative Neoplasms. The classical model of mutation acquisition in cancer (left panel) corresponds to the linear and sequential acquisition of mutations one after the other in sub-clones derived from each other. However, several studies of MPN clonal architecture found more complex paths of clonal evolutions. The driver mutation (in red) may be acquired first (center panel), followed by the acquisition of additional mutations in a branching evolution defining clones that diverge at several steps [17, 39, 40, 53]. Alternatively, the driver mutation may be acquired in a preleukemic clone that already carries a mutation in genes such as *TET2*, *DNMT3A* or genes of the spliceosome (right panel) [19].

The evolution of MPNs spans over many years between the acquisition of the driver mutation and the clinical manifestations leading to disease diagnosis, but also between the chronic phase and the secondary evolution towards myelofibrosis or acute leukemia observed in some patients. As noted above, this second phase is often associated with the acquisition of secondary or additional mutations. Two models of mutation acquisition were described in different malignant diseases. The classical model corresponds to the linear and sequential acquisition of mutations one after the other in sub-clones derived from each other. However, secondary mutations, although all acquired downstream of a driver mutation, can also develop in a branching evolution pattern with clones that diverge at several landmarks (Fig. 2). A consequence of such branched tumor evolution is intra-tumor heterogeneity (ITH) that defines the coexistence of molecularly and phenotypically distinct subclones within a tumor. Morphological heterogeneity has been long recognized by pathologists in solid tumors [34]. Those initial discoveries were substantiated by several groups with orthogonal techniques, which provided evidence for intra-tumor diversity across multiple cancer types. In a seminal work examining B-ALL, an aggressive hematological malignancy, fluorescence in situ hybridization and/or copy number alterations detected by SNP arrays studies uncovered that these hematopoietic malignancies are not monoclonal diseases but manifest as a collection of genetically distinct subclones [35]. This was also demonstrated in AML using whole genome sequencing [36, 37]. It was only much later that further evidence of the heterogeneity of the different clones present within the same tumor could be demonstrated in AML by comparing the transcriptomes of individual cells within the same samples [38]. Until recently, the study of MPNs clonal architecture required cumbersome and low throughput assays such as progenitor culture followed by colony punching or single cell plate sorting. New single-cell methods recently became available allowing to perform next generation sequencing (NGS) analysis covering a large number of genes in several thousands of individual cells. Such high throughput micro-fluidic approaches have recently shown that the clonal architecture in patients with MPNs is often complex and mostly of the branched type with numerous subclones co-existing in individual patients [39, 40]. It also appears that clones without a driver mutation but carrying mutations in genes classically associated with CHIP (*TET2*, *DNMT3A*…) can emerge in parallel to MPN cells, making the molecular profiles even more difficult to interpret. Indeed, the development of single cell techniques has subsequently revealed more precisely the complexity of these tumors at the genetic level.

## Reconstruction of MPNs clonal architecture at the genetic level

Indirect single-cell genotyping techniques such as progenitor colony genotyping allow the reconstruction of the phylogeny of MPNs [11, 12, 41]. However, due to the low number of colonies evaluable and the difficulty to sequence several genes, they don’t allow a reliable reconstruction and quantification of the complexity of tumor cells. As discussed above, to achieve an accurate understanding of the subclonal evolution during MPNs development, clonal heterogeneity must be studied at the single-cell level. The rise of high throughput single-cell DNA sequencing technologies allowed to efficiently reconstitute the clonal architecture of genetically complex tumors. Indeed, clonal hematopoiesis in individuals without germline predisposition is associated with older age and usually involves single mutations affecting *DNMT3A*, *TET2*, or *ASXL1* [6, 7]. Such mutations are also detected in myeloid malignancies with germline predisposition and can potentially modify disease progression and prognosis, making them important to track at the single cell level [42, 43]. In this field two important articles reported findings of single cells targeted genome sequencing in large cohorts of AML patients [39, 44]. To obtain a longitudinal view of disease development, Miles et al. studied 14 individuals with CHIP and 14 individuals with MPN, both considered as pre-stages of AML, as well as six MPN samples from patients who progressed to AML. Finally, to obtain information on the risk of relapse, AML samples before and after therapy were also included. In summary, the authors firmly established at the single-cell level that: (1) AML presents as an oligoclonal disease with a branched trajectory; (2) clonal evolution occurs in a clear order of events, with mutations in epigenetic factors preceding mutations in signaling genes; (3) signaling mutations in *RAS* and *FLT3* genes are independent and (4) clonal heterogeneity evolves over time, especially under environmental pressure such as treatment exposure. Similar work was performed in MDS on samples harboring several splicing mutations. Single cell DNAseq showed that SF3B1^K700E^ and SRSF2^P95H^ mutations occur in different cells [45]. Similarly, single cell DNAseq of paired chronic and transformed MDS samples revealed a patient-specific clonal evolution and enabled the assessment of co-mutations at single cell level. Also in MDS, Guess et al. discovered that modifications in the clonal architecture progress through distinct patterns, classified as static or dynamic, with dynamic clonal architectures having a more proliferative phenotype [46]. Analysis of chronic phase MPN samples showed comparable patterns according to the acquisition profile of the mutations. Indeed, in MPN the first acquired mutations always affect driver genes or epigenetic factors and *TP53* mutations are acquired late during disease evolution, often in a clone carrying a driver mutation or, when several *TP53* mutations are acquired, they occur in different cells [40]. Moreover, these high throughput techniques have also been able to show in rare cases of MPN with two driver mutations (JAK2^V617F^ and either *CALR* or *MPL*) that the mutations were present in distinct cells despite the differences in allele burden [47].

## Reconstruction of MPNs clonal architecture at the transcriptomic and epigenetic levels

In MPNs, the most recent techniques dedicated to tumor heterogeneity analysis allowed to simultaneously study single cell genotypes and transcriptomes. Indeed, A. Mead’s team used TARGETseq [48, 49] to compare the transcriptomes of JAK2^V617F^ cells and wild-type cells carrying additional mutations in genes regulating the spliceosome, the epigenome, or both. This strategy is derived from an improvement of the SmartSeq-2 technique allowing to detect mutations on full length RNA which was previously applied to Chronic Myeloid Leukemia (CML) [50]. This technology enabled A. Mead’s team to link the genotypes to transcriptional changes in different clones from the same environment. This approach not only showed different gene expression profiles by high resolution t-SNE in *JAK2* wild-type and mutated cells, but also when the *JAK2*-mutated cells carried additional mutations in *ASXL1*, *U2AF1* or *SRSF2*. Each genetic subclone clustered separately and showed transcriptional differences driven by specific pathways such as pro-apoptotic pathways in *JAK2* and *U2AF1* mutated clones, JAK-STAT signaling in *JAK2*^V617F^ homozygous clones or pathways implicated in leukemogenesis in *JAK2* and *SRSF2* mutated clones [48]. More recently, the same team compared the single-cell transcriptomes of *TP53* wild-type or mutated hematopoietic stem and progenitor cells (HSPCs) isolated from MPN patients in chronic phase or at time of acute transformation. An important result of this study was that *TP53*-mutated cells harbor an inflammation promoting transcriptional profile, potentially contributing to the transformation mechanism by modulating the tumor microenvironment [51]. Using a similar but distinct approach, D. Landau’s team performed the genotyping of transcriptomes of *CALR*-mutated cells, but also of cells carrying JAK2^V617F^, *SF3B1* and *NFE2* mutations [52]. These two approaches have demonstrated that different clonal populations within the same tumor develop different expression programs that will greatly impact their intrinsic behavior and adaptability to the environment [53, 54]. More recent studies combining at the single cell level the genotyping of specific genes with the analysis of chromatin accessibility showed that JAK2^V617F^ mutated HSPCs exhibited specific proinflammatory signatures with accessibility to DNA motifs binding NF-kB or JUN/FOS factors. This technique also allowed to highlight epigenetic modifications specific to mutated erythroid or megakaryocytic progenitors, demonstrating that the consequences of the mutations are different according to the differentiation state and cellular context [55]. A similar approach showed that *DNMT3A* mutations resulted in myeloid over lymphoid bias and in expansion of immature myeloid progenitors primed toward megakaryocytic-erythroid fate, and also demonstrated the dysregulated expression of lineage and leukemia stem cell markers with the preferential hypomethylation of polycomb repressive complex 2 targets [56].

Altogether these results strongly suggest that the complexity of the genetic, transcriptomic and epigenetic clonal architecture is a new concept that should be considered when following tumor evolution as it impacts not only disease initiation but also its evolution and response to environmental factors. As such heterogeneity affects treatment response, clonal complexity should be integrated as a clinically assessable prognostic element to better apprehend patient care in the setting of chronic malignancies such as MPNs.

## Mechanisms driving MPNs clonal evolution

The natural history of MPNs is marked by the acquisition of several mutations over long periods, with certain mutations having an impact on the clinical course as described recently during normal [57] as well as clonal hematopoiesis [58]. For several years, NGS techniques allowed to explore a large number of genes in a single analysis and thus to precisely define the molecular profile of patients’ malignant cells. Furthermore, the sequential repetition of this analysis over time during patients’ follow-up allows for the detection of clonal changes. Indeed, in some patients MPN clones remain very stable over time, with a number and allele burden of mutations that barely vary over several years. However, treatment intervention can shape MPN clones. For example, interferon alpha therapy may reduce the JAK2^V617F^ or *CALR* mutations variant allelic frequencies [59, 60], which reflects a reduction of the tumor clone [61]. Recent single cell studies showed that response to treatment was heterogeneous according to the genetic profiles of the subclones (homozygous or heterozygous) [62, 63]. In contrast, in some patients important increases in the mutations allele burden and/or appearance of new mutations that may precede clinical evolution are observed. The later may reflect clonal evolution probably linked to the evolution of the disease. Indeed, a recent study reported that patients experiencing clonal evolution during follow-up had a poor prognosis with shorter myelofibrosis-free survival, leukemia-free survival and overall survival [64]. Also, it has been shown that the clone responsible for leukemic transformation of MPN was often already present at a low level during the chronic phase and gradually overtook the other clones [39]. These results strongly suggest that clonal evolution is an important marker of disease phenotype change.

However, the mechanisms leading to the emergence of a particular clone among the others are not elucidated. It is acknowledged that cancer development in general follows Darwinian principles [53] and that clonal selection is guided by forces related to the microenvironmental context [65]. The mechanisms of clonal selection are still poorly understood but several factors may play a role such as cell metabolism, competition for nutrients, clone-to-clone interactions, and cellular microenvironment including soluble factors such as cytokines but also drugs administered to patients. Indeed specific inflammatory cytokines such as IL-1β and IL-13 have recently been shown to be a major factor favoring JAK2^V617F^-mutated cells clonal expansion and bone marrow fibrosis in mouse models [66, 67]. Mutations in *TP53* leading to inactive forms of the protein are identified in 15% of chronic MPNs and are considered key events in the transformation into secondary acute leukemia. However, these mutations by themselves are not sufficient to confer a clonal advantage to HSCs in normal conditions, whereas ionizing radiations or chemotherapy induce the expansion of *TP53*-mutated cells in vivo, highlighting the role of the environment in the selection process [68]. It has been shown that chemotherapy treatments favor the expansion of *TP53* mutated clones [69] preceding the development of secondary leukemias, confirming the role of external pressure in clonal selection. MPN patients require very long-term treatment with cytoreductive therapy to avoid thrombosis. The potential role of these treatments in the transformation of MPNs has been suggested for several molecules. It is well established that prior exposure to certain cytoreductive agents, including radioactive phosphorus (^32^P), as well as pipobroman, busulfan, and other alkylating agents historically used for the treatment of PV and ET, are associated with accelerated leukemia development [70, 71]. It has been suggested that MPN cells of patients on long-term hydroxyurea therapy may acquire or select for *TP53* mutations but the mechanism involved is not known [72]. A recent study on a cohort of 1500 patients described the clonal evolution of MPN patients under various cytoreductive treatments [73]. In this study, hydroxyurea treatment was significantly associated with an expansion of *TP53*-mutated clones, while interferon alpha therapy was associated with the expansion of *DNMT3A* mutated clones, as previously reported [74]. These results suggest a specificity in the selective pressure that may be imposed by a given drug on specifically mutated clones. Since a model of chronic infection showed that interferon gamma signaling was involved in clonal selection of *DNMT3A*-mutated cells [75], a similar mechanism could be suspected for interferon alpha. Recently, expansion of *TP53*-mutated clones was observed in vivo in some MPN patients treated with an MDM2 inhibitor [76]. In these patients, treatment discontinuation was accompanied by a reduction in the allele burden of *TP53* mutations, supporting the idea that this drug exerts a direct selective pressure. An additional in vitro study using cultured *TP53* mutated and non-mutated CD34+ progenitors derived from patients with MPN clearly established that the exposure to an MDM2 inhibitor was directly responsible for the clonal selection of *TP53*-mutated cells [40]. Mechanistically, it can be hypothesized that the origin of the selection is probably related to the resistance of *TP53*-mutated cells to the MDM2 inhibitor-induced senescence, due to a potential loss of function or dominant-negative effect of heterozygous mutations as suggested in cellular models [77].

## Conclusion

MPNs depend on the acquisition of mutations both for disease initiation (driver mutations) and its secondary evolution (additional mutations). However, these diseases are very heterogeneous with patients harboring either very simple or very complex molecular profiles. Dissection of intra-clonal heterogeneity by new single-cell sequencing techniques seems essential to better understand the tumor complexity involved in disease evolution and treatment response. It appears that clonal evolution constitutes an important step in the worsening of MPNs phenotypes, but the precise mechanisms involved in such clonal remodeling remain poorly understood. As illustrated by the studies of *TP53* mutations selection, there are now strong arguments suggesting that the treatments administered to patients with MPNs during the long chronic phase of the disease can actively shape clonal fitness and evolution. Further studies are needed to assess whether other high-risk molecular mutations can be selected by the different molecules used in the treatment of MPNs but also by other environmental events. Finally, it is also important to assess whether interferon alpha has the ability, as shown for JAK2^V617F^ mutations, to reduce the expansion of the most deleterious clones.
